# A Case of Uveitis and Hypothyroidism Following Pembrolizumab Therapy in a Patient With Plantar Malignant Melanoma

**DOI:** 10.7759/cureus.73530

**Published:** 2024-11-12

**Authors:** Hitomi Sugino, Mayu Kitamura, Manabu Yoshioka, Natsuko Saito-Sasaki, Yu Sawada

**Affiliations:** 1 Dermatology, Kyushu Rosai Hospital, Kitakyushu, JPN; 2 Dermatology, University of Occupational and Environmental Health, Kitakyushu, JPN

**Keywords:** hypothyroidism, irae, melanoma, pembrolizumab, uveitis

## Abstract

Pembrolizumab is a PD-1 inhibitor that has improved survival in melanoma patients but can cause immune-related adverse events (irAEs) such as hypothyroidism and uveitis. Although these conditions rarely occur together, we present a case of a 54-year-old female with plantar malignant melanoma who developed hypothyroidism after the second cycle of pembrolizumab and then uveitis after the fifth cycle. Hypothyroidism was treated with levothyroxine, and uveitis was managed with corticosteroids. This case highlights the importance of careful monitoring and multidisciplinary management of these adverse events during immunotherapy.

## Introduction

Malignant melanoma is an aggressive neoplasm arising from melanocytes, and its prognosis can be poor, particularly in cases of advanced or metastatic disease [1]. Traditionally, the treatment of advanced melanoma was limited, but the advent of immune checkpoint inhibitors (ICIs), such as pembrolizumab, has markedly improved survival outcomes. ICIs function by blocking immune checkpoints such as PD-1, thereby allowing the immune system to target and destroy cancer cells more effectively [2]. While these therapies have revolutionized melanoma treatment, their efficacy is not universal, and some patients do not respond. Moreover, ICIs are associated with a spectrum of immune-related adverse events (irAEs), which pose new challenges for clinicians [3].

Uveitis, an ocular irAE, is relatively rare but can be sight-threatening if not promptly diagnosed and treated [4]. Other common irAEs include endocrine disorders, with hypothyroidism being one of the most frequently reported [3]. However, the simultaneous occurrence of uveitis and hypothyroidism is exceedingly rare, and reports discussing these conditions in conjunction remain scarce in the literature. The clinical management of such overlapping irAEs requires careful consideration to balance effective ICI treatment with the mitigation of adverse effects.

In this case report, we present a patient who developed skin symptoms, hypothyroidism, and uveitis in sequence during pembrolizumab immunotherapy for plantar malignant melanoma. We also discussed the possible connection between these irAEs and emphasized the importance of careful monitoring for ocular complications as skin and thyroid issues emerge.

## Case presentation

**Figure 1:**
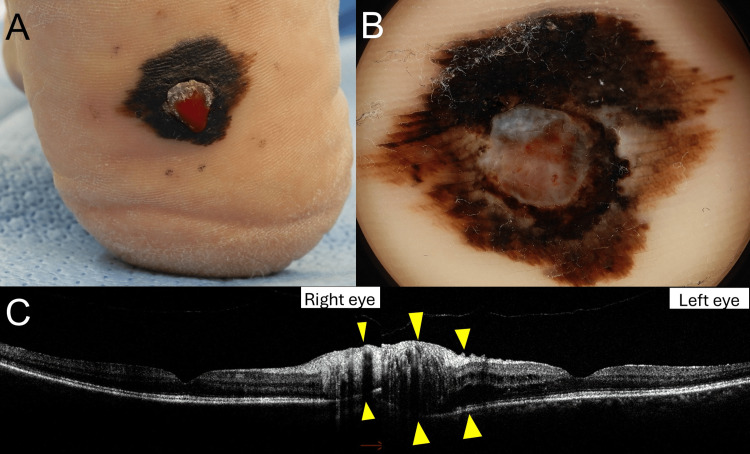
Skin tumor and eye examination (A) Clinical presentation of the right plantar malignant melanoma, showing a 24×18 mm irregularly bordered black lesion with a central 5 mm ulcerated nodule. (B) Dermoscopic image of the melanoma, revealing a parallel ridge pattern, consistent with malignant melanoma. (C) Optical coherence tomography (OCT) of the left eye shows retinal swelling around the optic nerve, indicative of uveitis associated with pembrolizumab treatment.

A 54-year-old female presented with a gradually enlarging black skin lesion with ulceration on her right plantar, which she had noticed three years prior. On physical examination, a 24×18 mm irregularly bordered black lesion with a central 5 mm ulcerated nodule was identified on the heel (Figure 1A). A dermoscopy of the lesion revealed a parallel ridge pattern, highly suggestive of malignant melanoma (Figure 1B). CT imaging denied distant metastasis of the tumor. The patient underwent wide local excision of the primary lesion and sentinel lymph node biopsy. Histopathological examination confirmed superficial spreading malignant melanoma (pT3bN2aM0, Stage IIIc) with a BRAF V600E mutation. The patient was started on pembrolizumab 400 mg every six weeks as adjuvant therapy. Following the second cycle of pembrolizumab, the patient developed skin eruption, which was resolved by topical betamethasone butyrate propionate ointment. Before the third cycle of pembrolizumab treatment (on day 12), laboratory results showed elevated TSH levels (6.02 mIU/L) and decreased free T4 (0.51 ng/dL) indicating hypothyroidism. Levothyroxine replacement therapy was initiated, and thyroid function stabilized under the supervision of an endocrinologist.

After completing the fifth cycle of pembrolizumab (on day 26 of treatment), the patient recognized photophobia, eye pain, and reduced vision. The ophthalmologic evaluation revealed severe inflammation in the anterior chamber of both eyes, with posterior synechiae observed in the left eye. Optical coherence tomography (OCT) showed retinal swelling around the optic nerve, more prominent in the left eye (Figure 1C). The patient experienced eye symptoms (ocular pain and photophobia) two weeks after the final dose of pembrolizumab, prompting the initiation of steroid eye drops. Three days later, a one-time sub-Tenon’s steroid injection was administered, and symptoms improved within another two weeks. We graded the adverse event of uveitis as grade 3 according to CTCAE criteria. For melanoma, the patient is currently under observation without active treatment. For uveitis, the patient has monthly follow-ups and continues steroid eye drops as needed.

## Discussion

ICIs, particularly PD-1 inhibitors, are known to induce a variety of irAEs. While thyroid dysfunction is one of the most common endocrine irAEs, uveitis is comparatively rare. The simultaneous occurrence of hypothyroidism and uveitis is even less frequently documented. Both conditions are immune-mediated, with PD-1 blockade causing the immune system to lose self-tolerance and attack organ tissues, including the thyroid and eyes.

The pathophysiological association between these irAEs may involve shared cytokine pathways. By inhibiting the PD-1 pathway, ICIs enhance the immune system's ability to attack cancer cells but also disrupt immune tolerance, leading to autoimmune responses against normal tissues, including the thyroid gland and the uveal tract [5]. This immune dysregulation is likely driven by a combination of cytokine imbalances, T-cell activation, and autoantibody production. Cytokines such as IL-17 and IL-23, which play critical roles in immune-mediated inflammation, may contribute to the development of both autoimmune thyroiditis and uveitis [6-8]. IL-17 promotes inflammation in both the thyroid and the uveal tract. Additionally, inflammatory cytokines like IFN-γ may exacerbate tissue damage in these organs [6]. These shared immune pathways suggest a complex interplay between ICI, cytokine dysregulation, and autoimmunity, which may underlie the simultaneous occurrence of these conditions.

For advanced melanoma, current treatment strategies primarily include ICIs and, for patients with BRAF mutations, combination therapies of BRAF and MEK inhibitors. Key ICIs include PD-1 inhibitors (pembrolizumab and nivolumab) and CTLA-4 inhibitors (ipilimumab), used either alone or in combination, and are also effective as adjuvant therapy to prevent recurrence post-surgery [9]. For ICI-induced hypothyroidism, levothyroxine (thyroid hormone replacement) is administered, with regular blood tests to monitor thyroid function [10]. ICI-induced uveitis is managed with steroid eye drops or sub-Tenon injections, and in severe cases, systemic steroids may be necessary [11]. Depending on symptom severity, ICI treatment may be continued or temporarily paused.

ICIs, especially PD-1 inhibitors, are effective for advanced melanoma but can cause immune-related side effects, including hypothyroidism and the less common uveitis. These side effects may arise from shared immune pathways, where cytokine imbalances and T-cell activation contribute to inflammation in both the thyroid and eyes. Management includes thyroid hormone replacement for hypothyroidism and corticosteroids for uveitis, with ICI treatment adjustments based on symptom severity.

## Conclusions

We present a rare case of pembrolizumab-induced hypothyroidism followed by uveitis in a patient with plantar malignant melanoma. As the use of ICIs continues to expand, clinicians must remain vigilant for the potential co-occurrence of multiple irAEs, even when individual conditions are uncommon. Early diagnosis, interdisciplinary collaboration, and prompt intervention are critical to managing these complex cases and optimizing patient outcomes. This case also underscores the need for further research into the relationship between endocrine and ocular irAEs to better inform clinical management strategies.

## References

[1] Long GV, Swetter SM, Menzies AM (2023). Cutaneous melanoma. Lancet.

[2] Carlino MS, Larkin J, Long GV (2021). Immune checkpoint inhibitors in melanoma. Lancet.

[3] Darnell EP, Mooradian MJ, Baruch EN, Yilmaz M, Reynolds KL (2020). Immune-related adverse events (irAEs): diagnosis, management, and clinical pearls. Curr Oncol Rep.

[4] Rali A, Huang Y, Yeh S (2022). Cancer immunotherapy and uveitis: balancing anti-tumor immunity and ocular autoimmunity. Int Ophthalmol Clin.

[5] Dolladille C, Ederhy S, Sassier M (2020). Immune checkpoint inhibitor rechallenge after immune-related adverse events in patients with cancer. JAMA Oncol.

[6] Chi W, Yang P, Li B (2007). IL-23 promotes CD4+ T cells to produce IL-17 in Vogt-Koyanagi-Harada disease. J Allergy Clin Immunol.

[7] Amadi-Obi A, Yu CR, Liu X (2007). TH17 cells contribute to uveitis and scleritis and are expanded by IL-2 and inhibited by IL-27/STAT1. Nat Med.

[8] Pyzik A, Grywalska E, Matyjaszek-Matuszek B, Roliński J (2015). Immune disorders in Hashimoto's thyroiditis: what do we know so far?. J Immunol Res.

[9] Gorry C, McCullagh L, O'Donnell H (2023). Neoadjuvant treatment for stage III and IV cutaneous melanoma. Cochrane Database Syst Rev.

[10] Iwama S, Kobayashi T, Yasuda Y, Arima H (2022). Immune checkpoint inhibitor-related thyroid dysfunction. Best Pract Res Clin Endocrinol Metab.

[11] Rali A, Huang Y, Yeh S (2022). Cancer immunotherapy and uveitis: balancing anti-tumor immunity and ocular autoimmunity. Int Ophthalmol Clin.

